# Recurrent testicular torsion after orchidopexy

**DOI:** 10.11604/pamj.2015.20.190.6417

**Published:** 2015-03-02

**Authors:** Kostas Chondros, Nikolaos Chondros

**Affiliations:** 1 Department of Urology, University General Hospital of Heraklion, Heraklion, Crete, Greece

**Keywords:** Torsion, orchidopexy, testicle, subdartos pouch

## Image in medicine

A 16-year-old male presented with a sudden, severe right scrotal pain at rest, occurred within the last 2 hours. The patient had a history of right orchidopexy 12 months ago. There was no associated fever, injury, or sports activity. Clinical examination and color-Doppler ultrasound confirmed the diagnosis of testicular torsion, with the absence of intraparenchymal blood flow. The patient was submitted to emergent scrotal exploration, and a 360-degree spermatic cord intravaginal torsion was confirmed intraoperativelly (A). After detorsion and worm pad instillation to the testis, color improvement was observed and organ retention was decided. In addition, two small scars in the anterior area of the testicle were observed (B), suggesting previous orchidopexy with failure of absorbable suture fixation. Finally, orchidopexy with subdartos pouch was performed in order to secure the ipsilateral testicle and classical contralateral orchidopexy for metachronous torsion prevention. In conclusion, recurrent testicular torsion after previous surgical treatment failure is rather uncommon and clinical alertness is mandatory.

**Figure 1:**
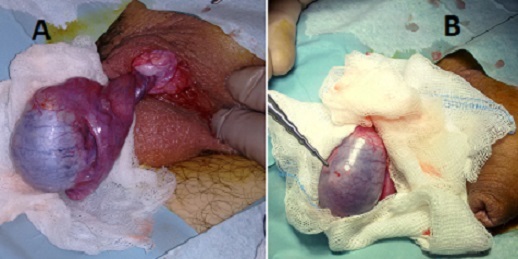
Intra operative image of emergent scrotal exploration. A) Confirmation of testicular torsion B) Scars of previous orchidopexy sutures

